# Focal colorectal uptake in ^18^FDG-PET/CT: maximum standard uptake value as a trigger in a semi-automated screening setting

**DOI:** 10.1186/s40001-016-0195-z

**Published:** 2016-01-09

**Authors:** Wolfgang Luboldt, Baerbel Wiedemann, Sebastian Fischer, Boris Bodelle, Hans Joachim Luboldt, Frank Grünwald, Thomas J. Vogl

**Affiliations:** Department of Radiology, Johann Wolfgang von Goethe University Hospital, Frankfurt, Germany; Multiorgan Screening Foundation (www.multiorganscreening.org), Munich, Germany; Institute of Medical Informatics and Biometry, University Hospital, Dresden, Germany; Department of Nuclear Medicine, Johann Wolfgang von Goethe University Hospital, Frankfurt, Germany

**Keywords:** PET/CT, Colon, Colorectal cancer, Polyp, Screening, Multi-organ screening

## Abstract

**Background:**

Focal colorectal uptake in ^18^FDG-PET/CT may be associated with a malignancy and can be quantified. This provides the basis for an automatic trigger threshold above which cases are flagged for colonoscopic evaluation and below which for individual assessment.

**Purpose:**

To determine the lowest maximum standard uptake (SUV_max_) in colorectal cancer that could be used as a threshold to trigger endoscopic evaluation and to evaluate whether the SUV_max_ needs to be further normalised to a priori known extrinsic factors.

**Methods:**

The SUV_max_ was measured in 54 colorectal carcinomas and correlated with gender, age, blood glucose level, injected activity, body mass index and time to scan using *t* test or correlation coefficients (Pearson or Spearman, according to distribution).

**Results:**

There was no correlation between SUV_max_ and any of the extrinsic factors mentioned above. The lowest SUV_max_ value was 5 [mean ± SD (range): 11.1 ± 4.8 (5.0–24.6)].

**Conclusion:**

In contrast to most other screening techniques, semi-automation in colorectal screening seems possible with PET/CT. This opens the door for further study into the feasibility of automated screening. Independent from extrinsic factors, an SUV_max_ ≥5.0 in a focal colorectal uptake in ^18^FDG-PET/CT should automatically trigger for endoscopic evaluation, if not contraindicated. Cases with SUV_max_ <5 should be assessed individually before referral for endoscopy. Thus, more interpretation time could be spent on those cases with a lower uptake and more ambiguous diagnosis.

**Electronic supplementary material:**

The online version of this article (doi:10.1186/s40001-016-0195-z) contains supplementary material, which is available to authorized users.

## Background

^18^FDG-PET/CT is widely used in the detection and monitoring of most cancer entities. It provides images of malignant cell proliferation via the glucose uptake fuelling it thus combining glucose metabolism data with morphology. It is already successfully used for staging, restaging and follow-up, changing therapeutic management in up to 36 % of cases [1] and avoiding future tests in up to 91 % [2]. It was proposed for screening as early as 1997 [3] closely in line with CT colonography [4] and MR colonography [5]. The technique has progressed since that time and continues to do so. The only organs that remain a diagnostic challenge for ^18^FDG-PET/CT are the colon, breast, stomach, urinary tract and the carcinomas for which there are specific tumour markers [prostate-specific antigen (PSA), alpha fetoprotein (AFP), calcitonin, chromogranin A].

There have been no large studies to compare PET/CT with colonoscopy with respect to accuracy in the screening of colorectal cancer and its precursors.

PET/CT has three main advantages over colonoscopy and CT/MR colonography:

1. It is completely non-invasive—it avoids cleansing and distension of the colon and as such is more readily accepted than colonoscopy by the population.
2. It allows for early detection of extra-colonic diseases, a valuable side-benefit.
3. Its analysis can be more easily automated due to the 3D digitisation of glucose metabolism with high contrast between focal accumulation and normal distribution.

The latter advantage requires the definition of a *threshold* to automatically trigger further diagnosis, by endoscopic evaluation in this case. In contrast to a cut-off, which separates benign and non-benign and thereby defines the outcome for both sides, a threshold value automatically triggers the outcome only for the group above the threshold. Individual and subjective analysis is then applied to those cases that fall below the threshold. This is already used in PSA screening in which transrectal palpation and/or ultrasound determines the decision to investigate PSA-negative carcinomas [6]. Safeguards must be devised for the group below the threshold, in order to minimise false negatives, in particular to facilitate the detection of small tumours.

The maximum standard uptake value (SUV_max_) is currently the most promising candidate to trigger further diagnosis and therapy as it reflects tumour vitality. Thus, the SUV is used to refine chemo- and radiation therapy according to vitality—the rationale behind interim staging with PET/CT [7, 8].

A threshold used to trigger colonoscopic evaluation for the majority of cancers should be independent of extrinsic effects. Thus, the purpose of the study was (a) to evaluate whether the SUV_max_ requires further normalisation and (b) to determine the lowest SUV_max_ to warrant automated referral for endoscopy.

## Methods

In a retrospective study approved by the Institutional Ethics Committee, patients with histologically proven colorectal cancer imaged with ^18^FDG-PET/CT before the onset of therapy were retrieved from a database search. Any patients with histologically proven colorectal cancer but a negative PET/CT would thus also have been included in the study.

PET/CT was performed 75 ± 14 min after injection of 329 ± 46 MBq FDG on a 16-slice PET/CT (Biograph 16, Siemens Medical Solutions) from the skull base through to the mid-thigh in 7–8 table positions each of 3-min duration. For attenuation correction, a low-dose (<1 mSv) CT was performed with 10 mAs, 120 kV, 16 × 1.5 mm collimation, 0.42 s tube rotation time and 6 mm/s table feed. CT images were reconstructed with 2.5-mm-thick slices. PET images were iteratively reconstructed using ordered subset expectation maximisation (OSEM) with 6 iterations, 4 subsets, 5 mm full width at half maximum (FWHM) smoothing and 168 × 168 reconstruction matrix for 70-cm gantry.

The SUV_max_ was measured in the colorectal tumour. The SUV_max_ is normalised for injected activity per body weight according to the formula: SUV_max_ = maximum VOI activity (Bq/ml)/dose injected per patient’s weight (Bq/g) with g = ml for a tissue density of 1 g/ml.

The association of SUV_max_ with T-stage, gender, age, blood glucose level, injected activity and time to scan (distribution phase) was analysed using *t* test or correlation coefficients (Pearson or Spearman according to distribution). Statistical analysis was performed using the standard software package SPSS Inc., version 16.0, Chicago, USA.

## Results

**Fig. 1:**
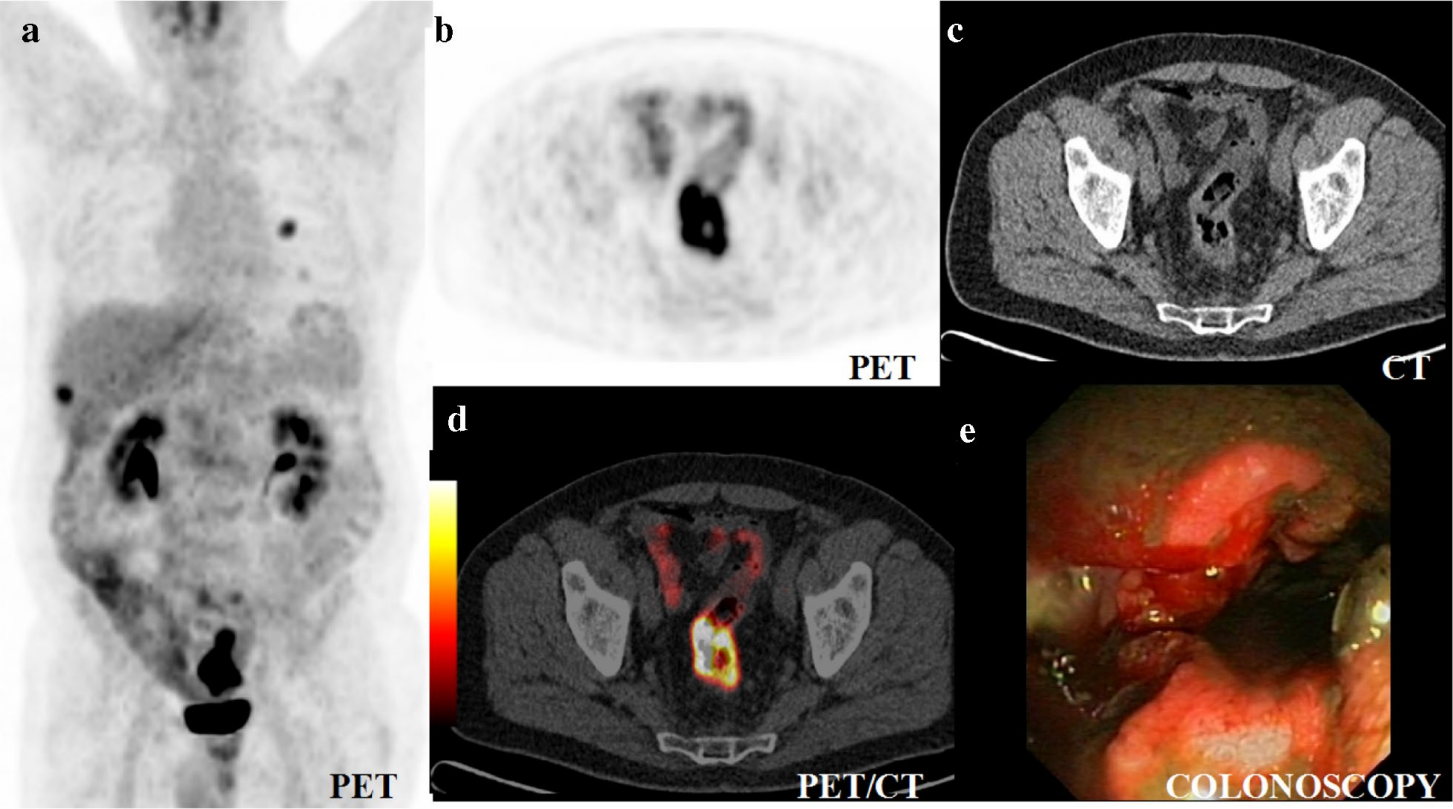
Colorectal cancer with solitary hepatic and lung metastases in the initial staging. Maximum intensity projection (MIP) of PET captured in the coronal projection of the 360°-rotation (**a**), axial slices of PET (**b**), CT (**c**) and PET/CT (**d**) and colonoscopy (**e**). The rotating MIPs of PET, here captured in coronal projection (**a**), enable clear depiction of colorectal cancer, here with a maximum standardised uptake value (SUV_max_) of 6.9, at the first view in contrast to CT (**c**) and colonoscopy (**e**) both of which are invasive, require bowel preparation and time consuming step-by-step analysis

**Fig. 2:**
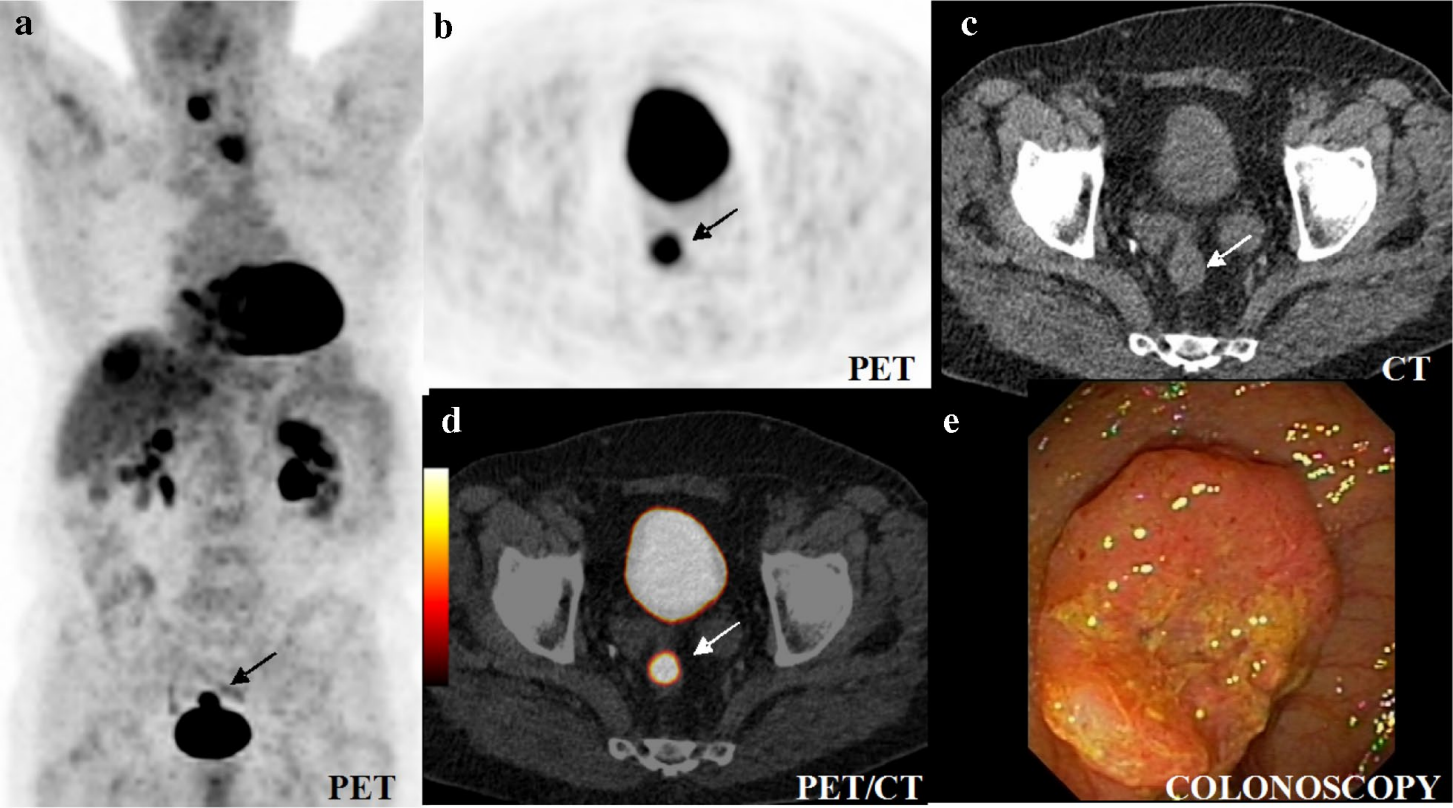
Colorectal cancer (*arrow*) as an incidental finding in the PET/CT restaging of oesophageal cancer with new lymph node and hepatic metastases. Maximum intensity projection (MIP) of PET captured in the coronal projection of the 360°-rotation (**a**), axial slices of PET (**b**), CT (**c**) and PET/CT (**d**) and colonoscopy (**e**). Note that the ^18^F-FDG-filled bladder can obscure carcinomas on the coronal maximum intensity projection (MIP) (**a**). Rotating MIPs or scrolling through axial PET slices (**b**) is mandatory to detect such carcinomas. The colorectal cancer, here with an SUV_max_ of 9.7, is clearly depicted on the axial PET images (**b**) in contrast to CT (**c**)

**Fig. 3:**
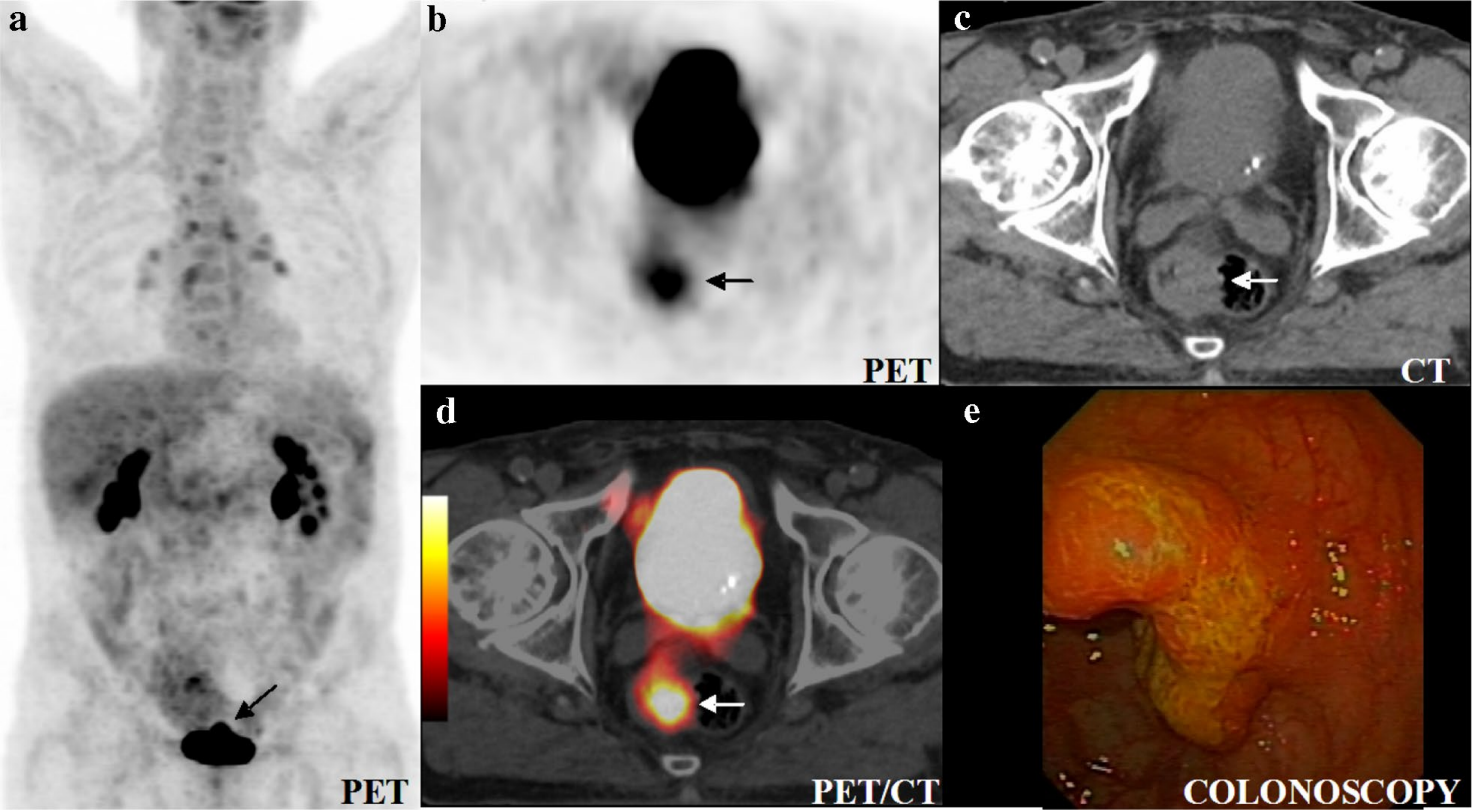
Colorectal cancer (*arrow*) in the initial staging. Maximum intensity projection (MIP) of PET captured in the coronal projection of the 360°-rotation (**a**), axial slices of PET (**b**), CT (**c**) and PET/CT (**d**) and colonoscopy (**e**). As in Fig. 2, rotating the maximum intensity projection (MIP) or scrolling through axial PET slices (**b**) is mandatory to detect carcinomas behind the urinary bladder. PET clearly depicts the colorectal cancer, here with an SUV_max_ of 6.1

Fifty-four patients (16 female, 38 male) aged 43–91 years (mean: 67 ± 10 years) were included in the study. Referring reasons for PET/CT were initial staging (*n* = 35) (Fig. 1), staging of another carcinoma with incidental detection of colorectal cancer as second cancer (*n* = 17) (Figs. 2, 3) and search for the primary cancer in cancer-of-unknown primary (CUP) syndrome (*n* = 2).

**Fig. 4:**
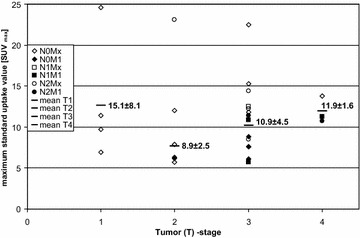
Maximum standard uptake value (SUV_max_) in relation to the TNM stage. All values were greater than 5, and there was no correlation between the SUV_max_ and the tumour stage in the 27 cases with available TNM stage data (Additional file 1: Table S1). [Note: The patient with the minimum SUV_max_ = 5 (Nr. 32 in Additional file 1: Table S1) is not included. The operation was performed outside the TNM stage and the data were thus not available]

In the tested ranges, there was no correlation between SUV_max_ and the extrinsic factors listed in Additional file 1: Table S1 [SUV_max_ vs. age (Pearson correlation coefficient = 0.074), SUV_max_ vs. body mass index (Pearson correlation coefficient = 0.148), SUV_max_ vs. injected activity (Pearson correlation coefficient = 0.185), SUV_max_ vs. glucose level (Spearman correlation coefficient = 0.047) and SUV_max_ vs. time to scan (Spearman correlation coefficient = −0.004)]. The SUV_max_ did not significantly differ among the various T-stages (all *p* values >0.05) (Fig. 4). The lowest *p* value was 0.07 between stages T2 and T4.

The lowest SUV_max_ value was 5 [mean ± SD (range): 11.1 ± 4.8 (5.0–24.6)] (Additional file 1: Table S1).

## Discussion

Semi-automation in colorectal screening seems feasible with PET/CT. A focal colorectal FDG accumulation with SUV_max_ ≥5 should automatically trigger a referral for colonoscopy leaving the cases with SUV_max_ <5 for individual interpretation. Even if the SUV_max_ varies with scanner type, the semi-automation approach seems robust; a possible scanner-related shift of this threshold value will be compensated by subjective interpretation as for any other focal colorectal uptake below the trigger threshold.

### Normalisation to a priori known extrinsic factors

We found no correlation between the SUV_max_ and a priori known extrinsic factors that could bias the SUV_max_ measurements (Additional file 1: Table S1). This suggests that the SUV_max_ does not need to be further normalised for these a priori known extrinsic factors. The lack of correlation between the SUV_max_ and the time to scan suggests that the SUV_max_ is independent of the time of scanning in the tested range 59–112 min (mean: 75 ± 14 min) after injection. Further study is needed using dynamic PET to test intra-individually at 30, 60, 90 and 120 min to ascertain which time interval between injection and imaging is optimal for the detection of malignant colorectal uptakes. It has recently been found that normalisation to the blood pool in the aorta provides a means of correcting for scan time dependence [9]. This requires further verification. The lack of correlation between the SUV_max_ and activity in the tested range 94–395 MBq (mean: 329 ± 46 MBq) suggests that the activity can be reduced, possibly independent of weight, to 200 MBq. This is of interest in situations where PET/CT may be offered to asymptomatic individuals instead of screening colonoscopy where endoscopy is contraindicated, refused or not possible to complete.

### Benefit vs. radiation risk of PET/CT

To be justified, the benefit of PET/CT in early detection must compensate for its radiation risk. With 200 MBq activity the total radiation exposure can be reduced to less than two times natural background radiation (200 MBq × 6.7 mSv/350 MBq [10] + 0.8 mSv [11] = 4.6 < 2 × 2.4 = 4.8 mSv, assuming a linear relationship between MBq and mSv if 350 MBq results in 6.7 mSv [10]). The dose issue is especially significant if PET/CT is used for screening before the onset of symptoms in healthy subjects. The purely hypothetical [12] and delayed radiation risk is compensated if PET/CT detects 3 % of the colorectal cancers which occur in 0.9 % of cases (colonoscopic prevalence in a screening setting) [13] (*x* % sensitivity × 0.9 % prevalence >0.005 %/mSv [14] radiation risk × 4.8 mSv radiation exposure; with *x* % >3 %). In this calculation of the minimum required sensitivity (*x* % > 3 %), the concurrent detection of extra-colonic cancer entities and advanced adenomas, as well as other serious conditions such as cardiovascular disease, were not taken into account thus underestimating the overall benefit of PET/CT. Furthermore, there is a 10- to 40-year delay [15] between the hypothetical induction and development of radiation-induced cancer; the natural history of a missed cancer, had PET/CT not been performed, is more severe than the natural history of a hypothetical and delayed induced cancer, had PET/CT been performed. Additionally, the benefit-to-risk ratio of PET/CT increases with age due to the decreasing radiation risk and increasing incidence of cancer. This is in contrast to colonoscopy, where the rate of complication increases with age, while the prophylactic meaning of a polypectomy decreases. This is especially relevant as colorectal screening is recommended for individuals up to 70 years of age [16].

### Determination of the optimum SUV_max_ threshold

In our study, we found the minimum SUV_max_ in 54 colorectal carcinomas to be 5 thus determining the threshold. There are only a few studies which showed an SUV_max_ lower than 5 for colorectal cancer. Sarikaya et al. [17] reported four carcinomas that were detected with SUV_max_ <4.5, of which three were mucinous. Peng et al. [18] reported a range in SUV_max_ from 3.1 to 28 which included two mucinous carcinomas. The low cellularity of mucinous carcinomas may explain the low SUV_max_. A meta-analysis regarding the SUV_max_ of colorectal cancer is not possible because the SUV_max_ was not always listed for each carcinoma. When SUV_max_ is used as the sole trigger, and not in combination with other factors, an SUV_max_ threshold of 5 would cover 96 % (215/224) of FDG-positive colorectal cancers (Figs. 1, 2, 3, 4) [17–31], leaving only 4 % of positive cases requiring individual interpretation.

### False negatives (FN) (carcinomas)

Besides the 4 % of PET-positive colorectal cancer cases that fall below the threshold, some cancer cases are completely PET negative. The rate of PET-negative *cancer* cases may be at least 5 % as suggested by studies looking at all patients who underwent PET/CT followed by colonoscopy within a short period of time [25, 30, 32]. On the other hand, the miss-rate of optical colonoscopy can be estimated at a worst case of 2.9 %, assuming that all 2.9 % of the so-called interval cancers occurring within 5 years of a negative colonoscopy were missed and not newly developed [33].

The issue of false negatives must be viewed in context. The vast majority of the German population currently does not come forward for screening due in large part to the invasive nature of colonoscopy. Between 2002 and 2008, 2,821,392 screening colonoscopies were performed across Germany which represents 15.5 and 17.2 % of all eligible men and women, respectively, from the age group 55–74 years [13]. Thus, approximately 80 % of the target group did not take advantage of the colonoscopy screening programme during this 6-year screening interval. Although the acceptance rate is higher in some other countries, such as the US, there is a widespread reluctance on the part of the population to come forward for colonoscopy-based screening. Shortcomings in alternative screening techniques must be balanced against the significant number of tumours which progress to a more advanced stage due to this very low acceptance of colonoscopy screening. PET/CT should not replace colonoscopy screening in the minority of individuals who assent, but provide an attractive alternative for the majority who refuse. Thus, if colonoscopy is refused, PET/CT needs to be compared with faecal occult blood test (FOBT) and not with colonoscopy.

### False positives (FP)

FDG-enriched stool in the caecum is the most common cause of false-positive FDG accumulation [11]. FDG excretion into the small bowel and accumulation in the caecum during the 60-min interval between injection and imaging may explain this observation. The typical location in the caecum in conjunction with centric distribution and air-typical CT values, which indicate air inclusions, helps to differentiate FDG-enriched stool from a wall-adherent eccentric mass. Although Van Heoij et al. [31] recently found that the SUV_max_ in 404 focal colorectal uptakes was significantly higher for cancer (*p* < 0.001) than for all other types of lesions (advanced adenoma, non-advanced adenoma and benign lesions), Keyzer et al. [34] showed that the SUV_max_ alone does not differentiate true- from false-positive colorectal FDG foci. The metabolic volume also failed to differentiate TP from FP [34, 35].

As the SUV_max_ in premalignant/malignant and physiological/benign colorectal FDG accumulation is indistinct, the clear separation (cut-off) between TP and FP seems to be unattainable with SUV_max_ alone. We therefore defined the trigger as automating the decision above a threshold only (semi-automated analysis). Given the relatively low prevalence of focal colorectal uptakes (3.6 %) but the relatively high risk of these being malignant or premalignant (68 %) [36], the benefit of maximising the sensitivity with semi-automated analysis seems to justify a lower specificity with more FPs. If colonoscopy is the worst consequence of an FP, these patients would not be disadvantaged compared to their outcome had they taken up the current screening programme [16, 37]. In comparison to colonoscopy, the 1.5 % rate of FP in PET/CT [36] with consecutive colonoscopy is far lower than the 26.5 % rate of false-positive polypectomies, several polypectomies per person not counted [13].

### Partial volume effects: a drawback of digitisation

Averaging within a volume pixel (voxel) of a finite edge length is a drawback of digitisation. Smaller lesions in the range of only view voxels might not be visible due to spatial and temporal averaging within one voxel (partial volume artefacts) [38]. The resultant blurring might reduce the overall contrast so that the lesion is not delineated. However, a very high uptake—the so-called hot spot phenomenon, as known from melanoma—might compensate for a larger voxel size and even depict lesions within the range of the voxel resolution [currently: 95 mm^3^ (=0.095 ml) based on 400 × 400 matrix reconstruction]. However, this potential inferiority in voxel resolution compared to optical colonoscopy might be compensated by a shorter screening interval (e.g. 5 years as for CT colonography [37]). This may be completely unnecessary when the long lead time of 10 years in the adenoma-to-carcinoma sequence is taken into account [39–42] and the fact that therapy in asymptomatic (lower stage) colorectal cancer is mostly curative. Furthermore, it must be emphasised that lower voxel resolution can easily be compensated for by a shorter screening interval, in contrast to a lower screening acceptance rate which cannot be compensated for.

### Extrapolation to advanced adenoma

There is some evidence that PET/CT failed to detect around half of cases with *advanced adenoma* [43]. The study was performed between 2000 and 2009 using now outdated PET and PET/CT technology. Since then, the spatial resolution has improved from 4.5 mm to almost 2 mm today, for example. In an interval screening programme, it is the accuracy of the programme and not of the single test which matters. Furthermore, the consequence of a missed tiny adenoma is unclear if the cancer can still be curatively resected at the consecutive screening, if indeed the adenoma develops to cancer at all. The mismatch in prevalence between advanced adenoma (6.4 %) and colorectal cancer (0.9 %) [13] suggests that not all advanced adenomas proceed to cancer. It is assumed that a patient with advanced adenoma at age 55–65 has a greater than 50 % chance of developing colon cancer [44]. The potential lack in screening sensitivity may be compensated by reducing the interval between examinations (for example from 10 to 5 years as proposed for CT colonography [37]), but a low screening acceptance rate cannot be compensated.

To date, we have neither included advanced adenoma nor correlated the FDG uptake with the KI 67 index as markers for proliferation. We measured FDG uptake versus TNM stage, however, and found no correlation (Fig. 4). Pending further study, we might extrapolate that the trigger SUV_max_ ≥5 is also valid for advanced adenoma, depending on the growth rate. The hypothesis that the SUV_max_ correlates with the growth rate seems correct; glucose provides the energy for proliferation and is supported by the relationship between pre-operative ^18^FDG uptake and epidermal growth factor receptor [45]. Also, ^18^FDG-PET detects all cancers in patients with familial adenomatous polyposis [46]. This is still speculative, however, pending a larger study. Recently, Na et al. [47] proposed an SUV_max_ = 5.8 as optimal cut-off to identify a malignancy or high-grade dysplasia but warned that colonoscopy should be performed above an SUV_max_ = 2.5 to avoid missing a malignancy or high-grade dysplasia. This is in line with the semi-automation we propose: an SUV_max_ ≥5 should automatically trigger a referral for colonoscopy leaving the cases with SUV_max_ <5 for individual interpretation.

### Case for PET/CT screening

A great deal of expertise and resources are currently invested in establishing, testing and improving *mono-organ* screening methods. Screening programmes are currently in place for the early detection of oncological, cardiovascular and metabolic diseases including prostate, lung, colorectal, ovarian [48] and breast cancer, as well as arteriosclerosis, aortic aneurysm and osteoporosis. PET/CT offers the possibility of replacing most *mono-organ* screening methods with a single *multi-organ* screening exam. A single PET/CT screening appointment lasting around 1 h promises to be more accepted, efficient, effective and safe than the combined organ-specific screening techniques currently in use.

In addition, PET/CT is a promising candidate for semi-automated analysis as it acquires digital data, in vivo, at the molecular level. In the context of colorectal cancer, this cannot be said of the subjective optical interpretation at the macroscopic level required for colonoscopy. Although laxative-free CT colonography [49] and PET/CT are both non-invasive and require no bowel preparation, PET/CT seems superior for multi-organ screening and semi- or potentially full automation of the analysis, as we have discussed.

## Conclusion

In contrast to most other screening techniques, semi-automation in colorectal screening seems possible with PET/CT. This opens the door for further study into the feasibility of automated screening. Independent of extrinsic factors, an SUV_max_ ≥5.0 in a focal colorectal uptake in ^18^FDG-PET/CT should automatically trigger endoscopic evaluation, if not contraindicated. This would improve the experience many individuals have during the screening process itself, as well as saving the time and cost of detailed interpretation of colorectal screening across the board. Only cases with SUV_max_ <5.0 should be referred for individual assessment.

## Additional file

10.1186/s40001-016-0195-z Demographic Data of Patients with Colorectal Cancer (n=54).

## Abbreviations

^18^FDG: 2-[F-18]fluoro-2-deoxy-d-glucose
PET/CT: positron emission tomography/computed tomography
SUV_max_: maximum standardised uptake value
MIP: maximum intensity projection
voxel: volume pixel
