# Supplementary material for: Focal colorectal uptake in 18FDG-PET/CT: maximum standard uptake value as a trigger in a semi-automated screening setting
Source: Eur J Med Res. 2016 Jan 9;21:2. doi: 10.1186/s40001-016-0195-z (PMC4706998; doi:10.1186/s40001-016-0195-z)
Supplement: Supplementary file 1 — 10.1186/s40001-016-0195-z Demographic Data of Patients with Colorectal Cancer (n=54). [file 40001_2016_195_MOESM1_ESM.doc]

| **TABLE S1. Demographic Data of Patients with Colorectal Cancer (n=54)** | | | | | | | | |
| --- | --- | --- | --- | --- | --- | --- | --- | --- |
| **Pat.**  **N°** | **TNM**  **stage** | **SUV_max_** | **Extrinsic parameters** | | | | | |
|  |  |  | Gender | Age [y] | BGL *  [mmol/L] | Activity  [MBq] | Body mass  index | Time to scan [min.] |
| **range** |  | **5.0-24.6** | **f:m** | **43-91** | **5-12** | **194-395** | **24-111** | **59-112** |
| **mean** |  | **11.1±4.8** | **16:38** | **67±10** | **6.3±1.7** | **329±46** | **71±22** | **75±14** |
| 1 | − | 8.9 | f | 86 | 5.88 | 225 | 55 | - |
| 2 | T4N2M1 | 10.7 | m | 43 | 5.72 | 250 | 87 | - |
| 3 | − | 13.3 | f | 63 | 5.38 | 214 | 64 | - |
| 4 | T3d N2(4/27) M1 | 11.4 | m | 64 | 11.60 | 354 | 37.6 | 71 |
| 5 | T3b N1(1/19) Mx | 12.5 | f | 59 | 5.7 | 312 | 59 | 105 |
| 6 | − | 20.5 | f | 56 | 5.80 | 363 | 94 | 62 |
| 7 | − | 8.8 | f | 71 | 5.70 | 340 | 78 | 82 |
| 8 | − | 6.4 | m | 71 | - | 319 | - | - |
| 9 | T2N0(0/12)Mx | 6.3 | f | 71 | 5.10 | 319 | 52 | 79 |
| 10 | T3b N1(2/13) M1 | 5.7 | m | 51 | 5.60 | 348 | 70 | 73 |
| 11 | − | 17.7 | f | 78 | 8.50 | 334 | 75 | 64 |
| 12 | − | 9.6 | m | 67 | 6.9 | 341 | 29.1 | 63 |
| 13 | − | 7.2 | m | 66 | 5.60 | 325 | 105 | 69 |
| 14 | − | 14.7 | m | 73 | 9.80 | 353 | 89 | 71 |
| 15 | − | 11.5 | m | 66 | 7.90 | 330 | 70 | 79 |
| 16 | T3aN0(0/14)Mx | 11.8 | m | 70 | 5.20 | 301 | 69 | 66 |
| 17 | T3aN0(0/21)M1 | 8.8 | m | 61 | 5.20 | 338 | 103 | 76 |
| 18 | T2N0(0/4)Mx | 7.9 | m | 70 | 5.7 | 340 | 90 | 60 |
| 19 | − | 20.5 | f | 77 | 5.30 | 391 | 81 | 90 |
| 20 | T1N0(0/14)Mx | 24.6 | m | 57 | 5.60 | 365 | 68 | 65 |
| 21 | T2N0M1 | 6.2 | m | 62 | 5.20 | 354 | 77 | 67 |
| 22 | T3aN2(7/20)Mx | 8.5 | m | 66 | 7.00 | 235 | 58 | 82 |
| 23 | T3aN2(7/20)Mx | 12.2 | m | 66 | 5.60 | 307 | 70 | 61 |
| 24 | T3cN2(1/10)Mx | 14.4 | m | 72 | 5.8 | 371 | 25.2 | 85 |
| 25 | T3cN0(0/14)M1 | 7.6 | m | 65 | 8.8 | 351 | 25.4 | 82 |
| 26  27 | T3bN0 (0/12)Mx | 6.1 | m | 74 | 7.2 | 332 | 24.1 | 85 |
|  | − | 13.7 | f | 77 | 6.80 | 395 | 95 | 91 |
| 28 | T2N2Mx | 23.1 | m | 67 | 5.80 | 340 | 105 | 70 |
| 29 | − | 14.5 | m | 60 | 4.60 | 313 | 70 | 67 |
| 30 | T4N1M1 | 11.3 | f | 53 | 5.80 | 362 | 70 | 69 |
| 31 | T3bN0(0/23)Mx | 22.5 | m | 69 | 4.90 | 379 | 80 | 97 |
| 32 | − | 5 | m | 58 | 4.70 | 369 | 68 | 63 |
| 33 | T2N0(0/13)Mx | 10.2 | m | 73 | 5.30 | 323 | 84 | 71 |
| 34 | − | 5.7 | f | 78 | 5.80 | 382 | 97 | 89 |
| 35 | − | 7.7 | f | 79 | 6.60 | 339 | 69 | 72 |
| 36 | − | 7.2 | m | 59 | 5.50 | 313 | 59 | 59 |
| 37 | T3NxMx | 7.6 | m | 91 | 5.44 | 194 | 72 | - |
| 38 | T4bN0(0/13)Mx | 13.8 | m | 59 | 5.50 | 289 | 68 | 81 |
| 39  40 | − | 5.1 | f | 69 | 8.70 | 312 | 74 | 86 |
|  | T3cN0(0/22)Mx | 15.3 | f | 89 | 5.60 | 355 | 59 | 112 |
| 41 | − | 6.1 | f | 59 | 5.10 | 375 | 62 | 73 |
| 42 | T1NxMx | 6.9 | m | 76 | 11.8 | 330 | 31.2 | 59 |
| 43 | − | 14 | m | 85 | - | 235 | 89 | - |
| 44 | T3N1M1 | 10.9 | f | 71 | 5.05 | 278 | 70 | - |
| 45 | − | 8.5 | m | 57 | 5.10 | 378 | 92 | 110 |
| 46 | − | 13.2 | m | 61 | 5.80 | 356 | 90 | 72 |
| 47 | − | 12.6 | m | 72 | 5.20 | 314 | 63 | 65 |
| 48 | T2aN0(0/8)Mx | 12 | m | 73 | 5.10 | 337 | 61 | 62 |
| 49 | − | 11 | m | 59 | 7.20 | 364 | 107 | 88 |
| 50 | T1N0Mx | 9.7 | m | 76 | 6.10 | 331 | 26.9 | 66 |
| 51 | − | 8 | m | 58 | 5.80 | 303 | 111 | 103 |
| 52 | T1N0Mx | 11.4 | m | 80 | 9.80 | 375 | 82 | 60 |
| 53 | − | 5.1 | m | 44 | 5.20 | 383 | 75 | 59 |
| 54 | − | 14.2 | m | 65 | 8.80 | 356 | 75 | 65 |

*blood glucose level
